# *In vivo* antiviral effect of plant essential oils against avian infectious bronchitis virus

**DOI:** 10.1186/s12917-022-03183-x

**Published:** 2022-03-07

**Authors:** Yu Zhang, Xiao-Yan Li, Bing-Sha Zhang, Li-Na Ren, Yan-Peng Lu, Jin-Wen Tang, Di Lv, Lu Yong, Li-Ting Lin, Zi-Xue Lin, Qin Mo, Mei-Lan Mo

**Affiliations:** 1 College of Animal Science and Technology, Guangxi University, Nanning, 530004 China; 2 Guangzhou Nasheng Biological Co., Ltd, Guangdong, 510650 China

**Keywords:** Plant essential oils, Infectious bronchitis virus, Cytokine, Inhibition, Immune response

## Abstract

**Background:**

Infectious bronchitis virus (IBV) leads to huge economic losses in the poultry industry worldwide. The high levels of mutations of IBV render vaccines partially protective. Therefore, it is urgent to explore an effective antiviral drug or agent. The present study aimed to investigate the in vivo anti-IBV activity of a mixture of plant essential oils (PEO) of cinnamaldehyde (CA) and glycerol monolaurate (GML), designated as Jin-Jing-Zi.

**Results:**

The antiviral effects were evaluated by clinical signs, viral loads, immune organ indices, antibody levels, and cytokine levels. The infection rates in the PEO-M (middle dose) and PEO-H (high dose) groups were significantly lower than those in the prevention, positive drug, and PEO-L (low dose) groups. The cure rates in the PEO-M and PEO-H groups were significantly higher than those in the prevention, positive drug, and PEO-L groups, and the PEO-M group had the highest cure rate of 92.31%. The symptom scores and IBV mRNA expression levels were significantly reduced in the PEO-M group. PEO significantly improved the immune organ indices and IBV-specific antibody titers of infected chickens. The anti-inflammatory factor levels of IL-4 and IFN-γ in the PEO-M group maintained high concentrations for a long time. The IL-6 levels in the PEO-M group were lower than those in prevention, positive drug, and PEO-L groups.

**Conclusion:**

The PEO had remarkable inhibition against IBV and the PEO acts by inhibiting virus multiplication and promoting immune function, suggesting that the PEO has great potential as a novel anti-IBV agent for inhibiting IBV infection.

**Supplementary Information:**

The online version contains supplementary material available at 10.1186/s12917-022-03183-x.

## Background

Infectious bronchitis virus (IBV) is a pathogenic avian coronavirus. Infectious bronchitis (IB) caused by IBV has brought serious economic losses to the poultry industry that result from the decline of weight gain, decreased egg production, eggshell quality, feed efficiency, false layers, and secondary bacterial infections [1, 2]. At present, vaccination is still the major efficient measure to prevent and control the disease. However, the IBV genome is prone to mutation and recombination, leading to more diversified and intricate serotypes that do not cross protect [3, 4]. Therefore, it is very important to explore alternative and efficient methods of IBV prevention or treatment. Natural plant compounds have been found to be effective against a variety of viral infections. Exploration and application of antiviral agents may be an alternative or complementary strategy for IBV vaccination.

Plant essential oils (PEO) are natural volatile aromatic substances extracted from various parts of plants as part of plant secondary metabolism [5]. PEO had been demonstrated to have good antiviral [6], antibacterial [7], anti-parasite [8], antioxidant [9], and immune-boosting properties [10], as well as low toxicity and virtually no residue in animals [11]. PEO and their constituents have been shown to have antiviral effects against animal coronaviruses [12–15]. It has been reported that Galanthus nivalis agglutinin was identified as a potent inhibitor of feline coronavirus (FCoV) by binding to the spike and membrane proteins of FCoV to prevent their attachment to the host cells [12]. In another study, the thymus vulgaris essential oil (TEO) was found to have remarkable anti-FCoV coronavirus activity, inhibiting viral replication at a concentration of 27 µg/mL and resulting in a significantly reduction of 2 log10 tissue culture infectious dose 50 (TCID_50_) /50 μL [13]. A previous study discovered that at very low concentrations, quercetin-7-rhamnoside inhibited viral activity by 50% against porcine epidemic diarrhea virus (PEDV) replication [14]. Grifthsin was also identified as a potent PEDV inhibitor that not only prevented infection by the cell-free virus but also virus transmission between infected and uninfected cells [15].

The use of plant extracts as an alternative or supplement to treatment or prevention strategy for IBV has not been extensively investigated. Only a few investigations have been performed to verify the anti-IBV activity of plant extracts [16–23]. A mixture of botanical oleoresins and essential oils was found to have virucidal activity against IBV infection both in vitro and in vivo [16]. *Houttuynia cordata* had an inhibitory effect on IBV in vitro and in vivo [17]. Additionally, some plant extracts, including *Sambucus nigra* [18]*, Mentha piperita, Thymus vulgaris, Desmodium canadense* extracts [19], *Astragalus polysaccharides* [20], *Glycyrrhiza radix* [21], and *Forsythia suspensa* [22] have shown effectiveness against IBV in vitro. Garlic extract successfully inhibited IBV in the replication phase in chicken embryos [23]. However, these studies have drawbacks, which have not focused primarily on PEO. Additionally, the above studies were mainly conducted in vitro [18–22], with few in vivo studies [23]. Furthermore, the difficulties frequently encountered when translating in vitro studies into in vivo treatments, suggesting that in vivo studies are necessary for practical treatment or prophylactic applications of different antiviral plant extracts.

In the present study, we investigated the in vivo antiviral effect of a mixture PEO of cinnamaldehyde (CA) and glycerol monolaurate (GML) against avian coronaviruses IBV. To the best of our knowledge, it is the first study to examine the effects of these plant preparations on IBV replication. We chose to study these two essential oil ingredients owing to their known antiviral properties. Cinnamon oil and CA significantly inhibited the growth of influenza virus and respiratory syncytial virus (RSV) [24]. Previous research has shown that CA promoted the growth of broilers, improved nutrition and biochemistry metabolism, enhanced immune function, and improved the quality of chicken meat [25, 26]. Cinnamon extract inhibits clathrin-dependent endocytosis and prevents the virus from entering host cells [27]. Thus CA is a potential anti-inflammatory, bioactive, and antiviral compound. Almost all medium-chain fatty acid monoglycerides have antiviral properties, particularly GML, which has potent antiviral properties and can inhibit virus infection and transmission, thus aiding in the prevention and treatment of viral diseases. According to the findings of a recent study, GML could inhibit the African swine fever virus (ASFV) in liquid conditions and reduce ASFV infectivity in feed [28]. Lauric acid inhibited the normal release of infectious vesicular stomatitis virus (VSV) by disrupting the viral proper assembly without affecting viral protein or nucleic acid synthesis [29]. Some of the viruses inactivated to some extent by GML include human immunodeficiency virus (HIV), measles, Herpes simplex-1, and influenza virus [30]. Combined, these findings suggested that CA and GML extracts may have broad antiviral properties.

Herein, the clinical efficacy evaluation, immune organ index, humoral immunity, and cellular immunity as well as the amount of viral RNA were used to assess the prevention and treatment effects of PEO. We sought to identify the antiviral effect of PEO containing CA and GML against IBV in vivo. Our results showed that the PEO has a good effect on inhibiting IBV infection, suggesting that the PEO has great potential as a novel anti-IBV agent.

## Results

### Clinical signs score

**Fig. 1:**
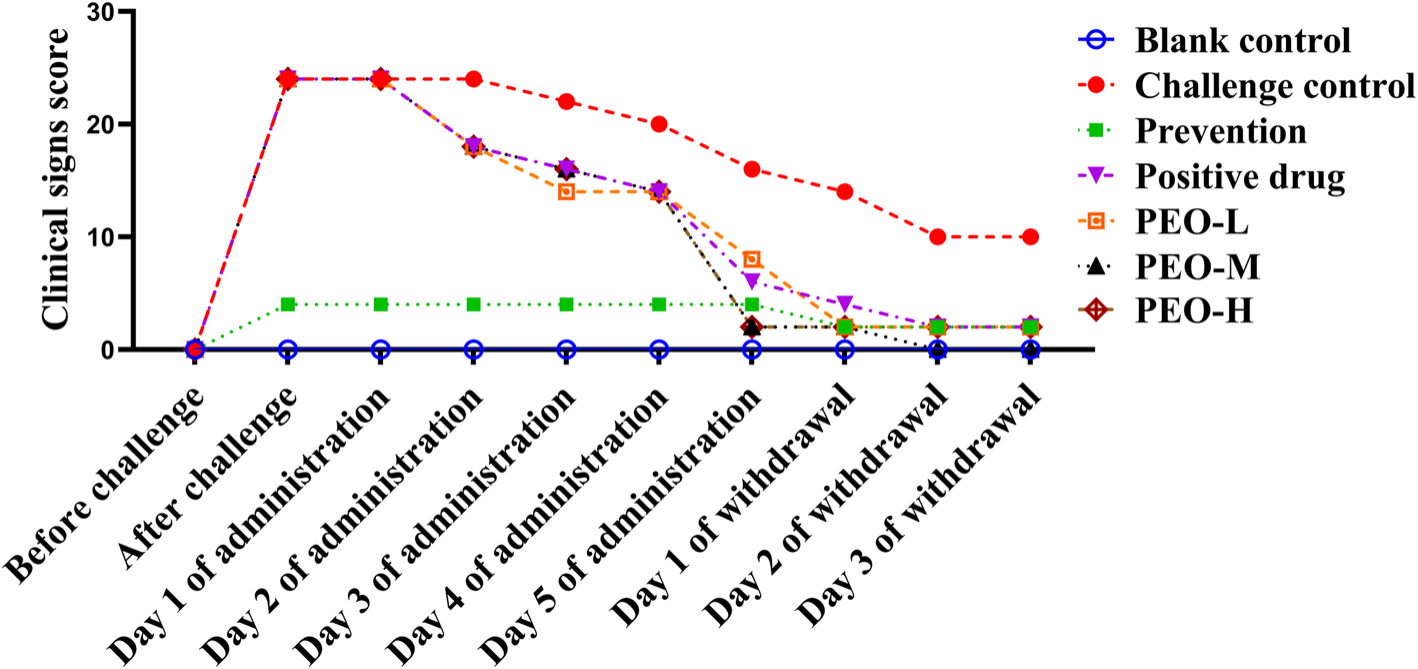
The clinical signs score of IBV-infected chicks treated with PEO in different periods. Note: Significant values were not marked in the figure

The clinical symptoms of IBV-infected chicks were observed and scored at various time intervals (Fig. 1). The symptom scores of the prevention group were consistently lower after the challenge of IBV. Birds in the challenge control group sustained higher symptom scores, while those in the PEO-L, PEO-M, PEO-H, and positive drug groups had lower scores as treatment progressed. At 2 days of administration, all drug groups’ scores decreased significantly (*p* < 0.01), with the challenge control group scoring significantly (*p* < 0.01) higher than the prevention and drug groups. At 5 days of administration, scores in the positive drug group and all PEO groups were significantly (*p* < 0.01) lower than that in the challenge control group. The PEO-M and PEO-H groups had the lowest symptom scores, which were significantly (*p* < 0.01) lower than the prevention, positive drug, and PEO-L groups. Two and three days after drug withdrawal, symptom scores were reduced to 0 in the PEO-M group, and to 2 in the prevention, positive drug, PEO-L, and PEO-H groups, all of which were significantly (*p* < 0.01) lower than those in the challenge control group.

### Clinical treatment effect

The chicks successively appeared depression, decreased appetite, increased water intake, frequent head shaking, hanging wings, coughing, mouth breathing, and tracheal rales at 3 days post-challenge (dpc). At 5 dpc, the morbidity reached a high level, accounting for more than half of the incidence. The positive drug and PEO groups were given drug treatment, respectively. The cure rate of each group was observed on the 7th day after drug administration (Table 1). The results indicated that the cure rates of prevention, positive drug, PEO-L, PEO-M, and PEO-H groups were 75.00%, 83.33%, 78.57%, 92.31%, and 91.67%, respectively. The cure rates of PEO-M and PEO-H groups were higher, at up to 92.31% and 91.67%, respectively. No death was witnessed in any groups during the experimental period. The protection rate of the prevention group was 60%. The therapeutic effect outperformed the preventive effect.

**Table 1:** Clinical prevention and treatment effects of PEO on IB

Groups	Number of animals	5 dpc		The 7 th day after administration		Number of cures	Cure rate (%)	Protection rate (%)
		Incidence number	Incidence rate	Incidence number	Incidence rate			
Blank control	20	——	——	——	——	——	——	——
Challenge control	20	15	75.00	14	70.00	——	——	——
Prevention	20	8	40.00	2	10.00	6	75.00	60.00
Positive drug	20	12	60.00	2	10.00	10	83.33	——
PEO-L	20	14	70.00	3	15.00	11	78.57	——
PEO-M	20	13	65.00	1	5.00	12	92.31	——
PEO-H	20	12	60.00	1	5.00	11	91.67	——

### Anti-IBV effects of PEO

**Fig. 2:**
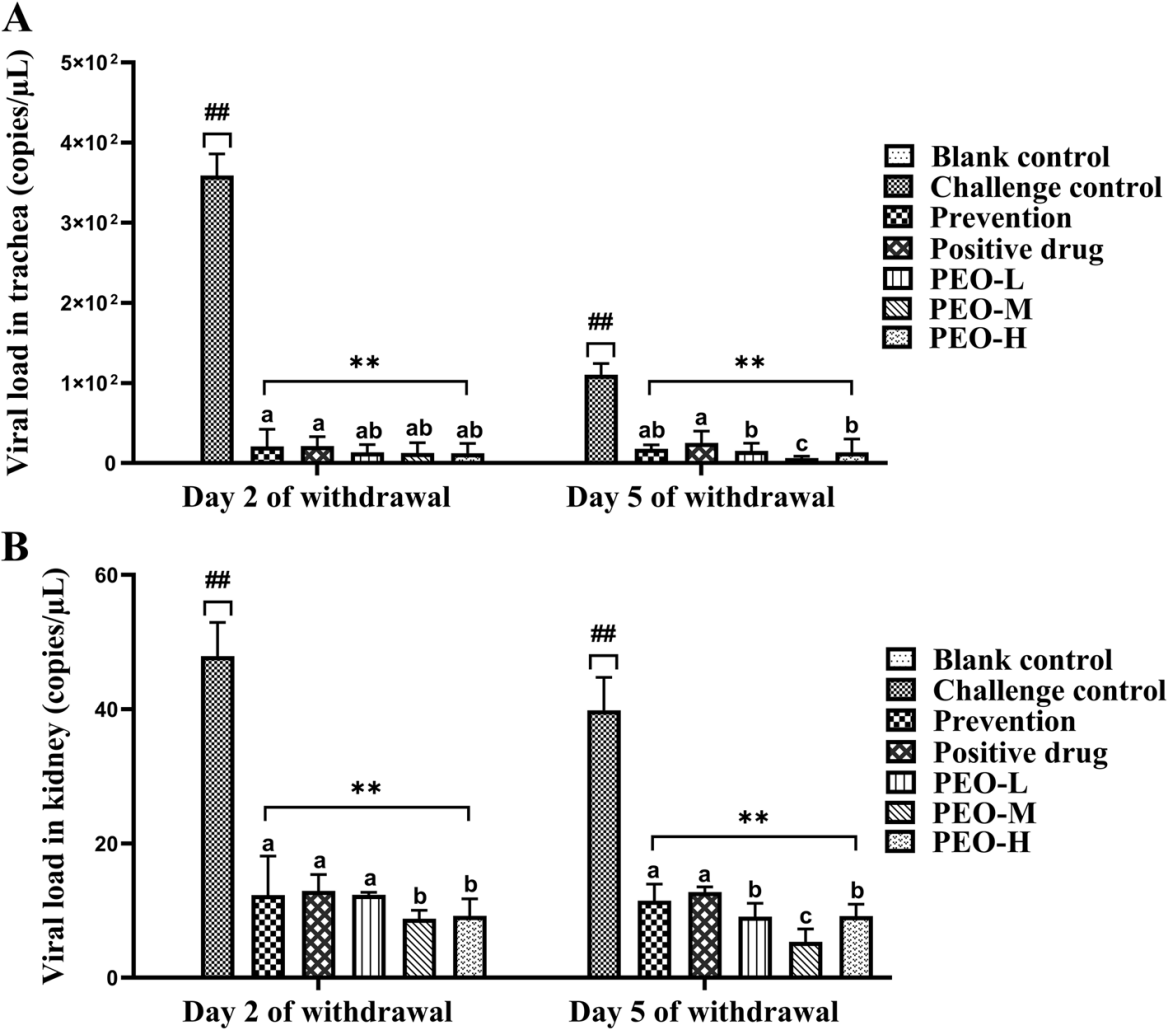
Viral loads in tracheas and kidneys. IBV RNA levels in tracheas and kidneys were evaluated on days 2 and 5 of drug withdrawal. **A** Viral loads in tracheas. **B** Viral loads in kidneys. Sample quantity *n* = 5; compared with the blank control group, the challenge control group showed # *p* < 0.05*,* ## *p* < 0.01; compared with the challenge control group, the prevention and drug groups (positive drug and different doses of PEO) showed *p < 0.05, ** *p* < 0.01. Different superscript letters indicated significant differences (*p* < 0.05)

To assure the inhibitory effect of PEO, the viral mRNA levels in the trachea and kidney of different groups were measured by real-time quantitative PCR (qPCR) at 2 and 5 days of drug withdrawal. No virus was detected in the trachea and kidney of birds of the blank control group. The mRNA expression levels of IBV in the trachea and kidney of the challenge control group were increased remarkably (*p* < 0.01) than those of positive drug and prevention groups (Fig. 2). In contrast to the challenge control group, the viral mRNA expression levels in the trachea and kidney of the chickens supplemented with PEO and the positive drug were reduced considerably (*p* < 0.01). Furthermore, after 5 days of drug withdrawal, the PEO-M group had lower viral loads in the trachea and kidney than the PEO-L and PEO-H groups, and the mRNA expression levels of IBV in PEO groups were significantly (*p* < 0.05) lower than those in the positive drug group. When compared to the challenge control, PEO supplementation resulted in a decrease in viral mRNA levels at the majority of time points.

### Effects of PEO on organs index

**Fig. 3:**
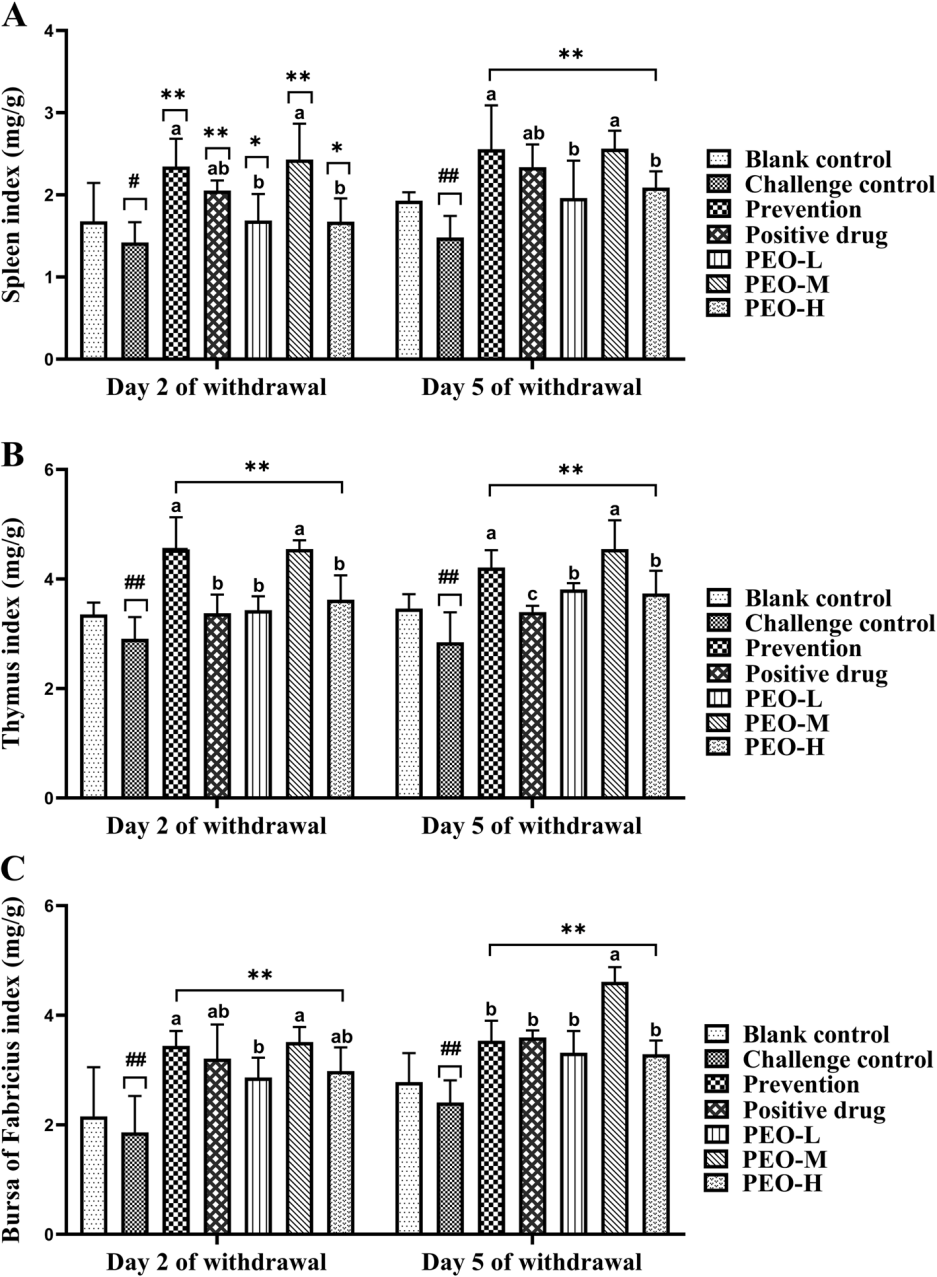
Effects of PEO on organ indices in chicks on days 2 and 5 drug withdrawal. **A** Spleen index. **B** Thymus index. **C** Bursa of Fabricius index. Sample quantity *n* = 5; compared with the blank control group, the challenge control group showed # *p* < 0.05*,* ## *p* < 0.01; compared with the challenge control group, the prevention and drug groups (positive drug and different doses of PEO) showed *p < 0.05, ** *p* < 0.01. Different superscript letters indicated significant differences (*p* < 0.05)

The spleen, thymus, and bursa of Fabricius were collected from 5 birds per group on the 2nd and 5th day after drug withdrawal and were weighed individually to calculate relative organ weights (Fig. 3). At 2 days after drug withdrawal, birds in the challenge control group had a reduced spleen index, which was significantly (*p* < 0.05) different compared to the blank control group. The spleen index of prevention, positive drug and PEO-M groups increased significantly (*p* < 0.01) compared with the challenge control group. The thymus and bursa of Fabricius indexes of the challenge control group were significantly (*p* < 0.01) lower than the blank group. Compared with the challenge group, the thymus and bursa of Fabricius increased in the prevention and drug groups, and the difference was very significant (*p* < 0.01). Furthermore, the prevention and PEO-M groups had higher organ indices than other drug groups. After 5 days of drug withdrawal, the spleen, thymus, and bursa of Fabricius indexes in the blank control group were significantly (*p* < 0.01) higher than those in the challenge control group. Spleen, thymus, and bursa indices were elevated in the prevention and drug groups, with highly significant differences compared with the challenge control group (*p* < 0.01). The organ index of the PEO-M group was higher than those of the positive drug group, PEO-L, and PEO-H groups.

### Antibody responses

**Fig. 4:**
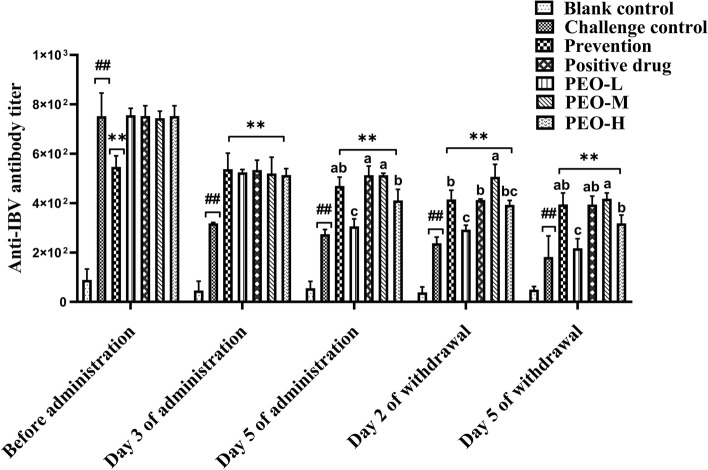
IBV-specific antibody titers in sera detected by indirect ELISA. Sample quantity *n* = 5; compared with the blank control group, the challenge control group showed ## *p* < 0.01; compared with the challenge control group, the prevention and drug groups (positive drug and different doses of PEO) showed ** *p* < 0.01. Different superscript letters indicated significant differences (*p* < 0.05)

To examine whether PEO has adjuvant effects on the humoral immune response in birds, antibody titers were determined by ELISA. As shown in Fig. 4, after challenge, the antibody titers of challenge control and drug groups increased notably and were significantly (*p* < 0.01) higher than those of the blank and preventive groups. Chickens in the challenge control group developed higher antibody titers than those in the blank control group 3 days after administration. On day 5 of administration, the antibody levels of the challenge control group were significantly (*p* < 0.01) higher than that of the blank control group. Moreover, the antibody titers in the different dose PEO groups, positive drug group, and preventive group were significantly (*p* < 0.01) higher than those in the challenge control group. The antibody levels in the PEO-M and positive drug groups were higher than those in other drug groups. After 2 and 5 days of drug withdrawal, the antibody titers in the prevention, positive drug, and PEO groups were significantly (*p* < 0.01) higher than those in the challenge control group, and the titers in the PEO-M and PEO-H groups were also significantly (*p* < 0.05) higher than that in the PEO-L group. During the experiment, compared with the blank control group, the challenge control group had significantly (*p* < 0.01) higher antibody titers. These results suggested that PEO could improve the humoral immune response in a dose-dependent manner.

### The effect of PEO on cytokine levels

**Fig. 5:**
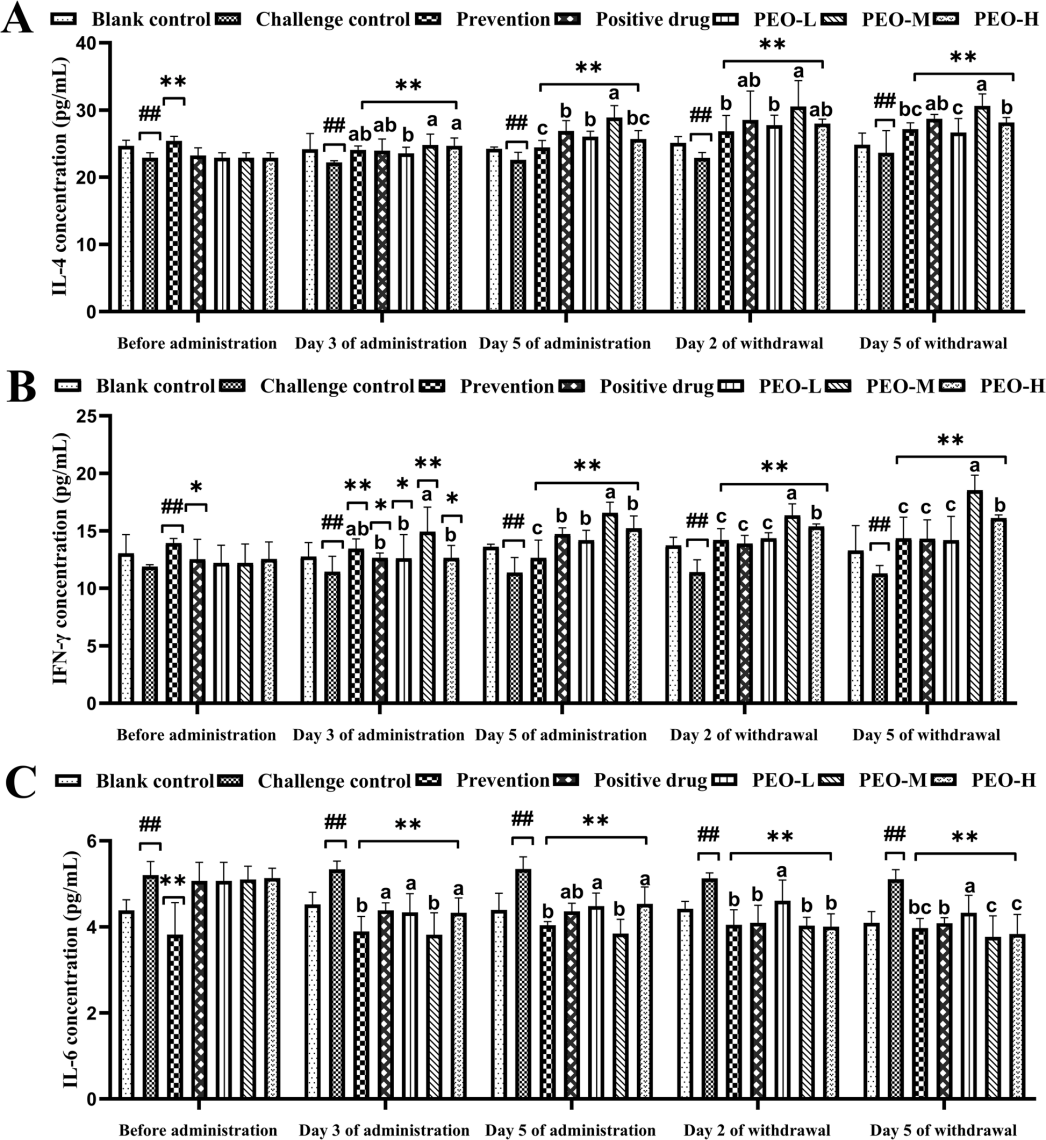
IL-4, IFN-γ, and IL-6 concentrations in the sera. **A** IL-4 concentrations. **B** IFN-γ concentrations. **C** IL-6 concentrations. Sample quantity *n* = 5; compared with the blank control group, the challenge control group showed ## *p* < 0.01; compared with the challenge control group, the prevention and drug groups (positive drug and different doses of PEO) showed *p < 0.05, ** *p* < 0.01. Different superscript letters indicated significant differences (*p* < 0.05)

To assess the effects of PEO on cytokines in IBV infected chickens, the levels of type II interferon IFN-γ, anti-inflammatory cytokine IL-4, and proinflammatory cytokine IL-6 were investigated during the experiments (Fig. 5). The results showed that the decreased IFN-γ and IL-4 production and exacerbated IL-6 expression were observed in the challenge control group compared with the blank control group. Before administration, expression levels of IFN-γ and IL-4 in the prevention group were significantly up-regulated than those of the virus infection group. On days 3 and 5 of administration, IFN-γ and IL-4 concentrations in the prevention and drug groups were significantly (*p* < 0.01 or *p* < 0.05) higher than those in the challenge control group. The same tendency was observed at 2 and 5 days of drug withdrawal. After administration, compared with the challenge control group, IL-6 expression levels in the prevention, positive drug, and PEO groups were notably diminished (*p* < 0.01). The levels of IL-6 in PEO groups were considerably downregulated alike to prevention and positive drug groups, and it was significantly decreased in the PEO-M and PEO-H groups at day 5 of drug withdrawal.

## Discussion

IBV is a Gamma coronavirus with multiple identified strains that continue to mutate and recombine, resulting in complex and diverse genotypes and serotypes of IBV. It is challenging to prevent and control IBV infection due to poor cross-protection between the vaccine and field strains. Thus, developing an effective antiviral therapy is a critical approach in combating IBV infection. We investigated the inhibitory effects of the synergistic blend of PEO including CA and GML on IBV in this study. Our findings indicated that the PEO used in this study has an antiviral effect on IBV within a range of doses. Especially PEO could significantly inhibit virus proliferation and improve immune function at 1.0 mL/L. To the best of our knowledge, this is the first application of PEO (including CA and GML) for the inhibition of IBV in China, establishing the groundwork for the development of anti-IBV infection drugs.

Previous studies have described various antiviral mechanisms of extracts from plant species against the avian IBV infection [17–19, 31–33]. The virucidal activity may be aimed at disrupting the viral membrane or interfering with viral envelope proteins involved in host cell attachment owing to their lipophilic nature [31]. These plant extracts have the potential to enter the viral membranes and cause disruption of the membranes. PEO and the phytochemicals in their composition interfere with viral replication and also benefit the host respiratory system through mucolytic and bronchodilatation [5]. Previous studies showed the terpenoid compounds 1,8-cineole, (-)-α- pinene and (-)-β-pinene have the potential to inhibit its interaction with the viral genomic RNA and break the IBV replication cycle by binding to the IBV nucleocapsid protein (N protein) [32, 33]. *Sambucus nigra* extract inhibits IBV in the early stages of infection and suggests that it does so by damaging the structure of the virus, possibly making it non-infectious [18]. Extracts of *Alium sativum*, *Houttuynia cordata*, and *Sambucus nigra* may be associated with direct inactivation of viral envelope structures that are required for adsorption to or entry into host cells or may lyse the IBV envelope [19]. The *Houttuynia cordata*-treated viruses were less infectious to chickens, suggesting the direct virucidal effect of *Houttuynia cordata* [17]. CA and GML have been shown to have antiviral potential against adenovirus (ADV) [34], influenza A/PR/8 virus [35], and ASFV [28]. The current study discovered that PEO had remarkably anti-IBV properties, such as reducing morbidity, inhibiting the IBV mRNA expression level, and improving cure rates in IBV infected birds. More specifically, in the case of antiviral effects, clinical scores in all PEO groups were at lower levels, which were significantly (*p* < 0.01) lower than that in the challenge control group (Fig. 1). The inhibition of IBV mRNA expression in PEO groups was remarkably (*p* < 0.01) lower than that in the challenge control group (Fig. 2). The PEO-M and PEO-H groups had higher effective and cure rates than the positive drug and preventive groups (Table 1). It was observed that PEO had potential antiviral activity within a certain dose range, which agreed with previous reports [17–20].

CA exerts an antiviral effect through direct inactivation, inhibition of viral replication and proliferation, and virus adsorption phases [35]. The mechanism of the GML anti-capsid virus is that lauric acid can modify the membrane protein of the virus. Because of its lipophilic nature, GML inserts into the vesicle membrane of the virus [28]. These effects probably cause the reduction or loss of replication ability of the virus, and the virus without replication ability acts as an antigen. The virus without replication capacity acts as an antigen and stimulates the animal to produce the corresponding antibody. The non-replicating virus acts as an antigen, stimulating the animal to produce the corresponding antibody, and thus acting as an antiviral agent [36].

To a certain extent, the level of organ index can reflect the strength of the body’s immune level, and it is a common indicator for evaluating the immune status of the body. The spleen is a vital peripheral immune organ that eliminates exogenous substances by stimulating lymphocyte proliferation and cytokine synthesis [37]. The thymus is an immune organ found in all vertebrates. Lymphatic stem cells develop and differentiate into mature T cells after entering the thymus. The bursa of Fabricius is a part of the central immune organ, which is a special lymphatic organ of birds, and it is of great significance to the establishment of poult immunity. Studies have shown that the spleen, thymus, and bursa of Fabricius can be used to assess the immune status of the body [38, 39]. The greater the absolute and relative weights, the better the cellular and humoral immune function of the body [40]. A previous study has found that using a specific combination of carvacrol, cinnamaldehyde, and *Capsicum oleoresin* on broiler chickens improved zootechnical performance response variables, had potent immunomodulatory effects, and improved intestinal integrity [26]. Our finding showed that the indices of spleen, thymus, and bursa of Fabricius increased in each dose of the PEO groups, which were consistent with other observations [41, 42]. It was discovered that the PEO promoted the development of chick immune organs, thereby improving the immune function of the body. The reason may be that the cinnamon oil, one of the ingredients in the PEO, is degraded into nutrients required by the body in the intestine after entering the body, and the nutrients enter the lymphocytes with blood circulation, thus promoting the proliferation of lymphocytes and promoting the development of the spleen, thymus, and bursa of Fabricius [43].

In the fight against infection, antibody level is an important indicator of humoral immunity. The humoral response is thought to be a crucial component of the immune response that protects against IBV. It has been reported that high antibody levels are associated with protection against IBV infection [44]. In addition, recent research has proved that essential oils can improve the cellular and humoral immune responses of chickens [45]. In this study, we found that administration of PEO increased serum anti-IBV antibody titers compared with the non-administered control group and the antibody levels in the PEO-M group were significantly (*p* < 0.05) higher than those in the preventive and positive drug groups. The titers in the PEO-M and PEO-H groups were significantly (*p* < 0.05) higher than those in the PEO-L group. These results showed that PEO could improve the humoral response in chickens, which was consistent with previous findings [46, 47].

An excessive inflammatory response, as a vital immune response of the organism, can cause a dramatic increase in cytokines in the organism, causing a disruption of the immune system and ultimately irreversible damage to the host. It has been reported that coronaviruses have developed strategies to evade the host innate mechanism by increasing the expression of proinflammatory cytokines and decreasing the production of type1 interferons [48, 49]. Furthermore, it has been established that proinflammatory cytokines play a critical role in IBV infection [50]. In the current study, in response to the heightened proinflammatory cytokine storm in IBV-infected chickens, PEO significantly enhanced the anti-inflammatory cytokines IL-4 and IFN-γ production when compared with the challenge control group. Meanwhile, we discovered that PEO has down-regulated the expression levels of proinflammatory cytokines IL-6 in chickens infected with IBV. Efficient coordination of pro- and anti-inflammatory cytokines is a necessary prerequisite for protecting the host from coronavirus infection [51]. IFN-γ is the only type II IFN found in birds and mammals, and it acts as a bridge between innate and adaptive immunity. IFN-γ regulates the maturation and differentiation of a variety of immune cells and activates T helper 1-type immune responses. Recent researches have distinctly defined the antiviral action of IFN-γ against viruses of various genetic nature including Newcastle disease virus (NDV) and Marek’s disease virus (MDV) [52, 53]. Chicken IFN-γ primarily induces MHC class I and class II molecules and mediates the production of nitricoxide, which is an important inhibitory mechanism for viruses [54, 55]. However, extensive future research is needed to underpin the mechanisms of IFN-γ induction of antiviral state. Th2 cells secrete IL-6 and IL-4, and IL-6 is closely associated with the inflammatory response. The present study showed that the PEO blend could promote IL-4 and IFN-γ expression while reducing the content of IL-6 in the serum of administered birds. These results suggested that PEO could improve the cell-mediated immune response.

In the present study, the potential antiviral activity of the PEO was investigated using the widespread and pathogenic Mass41 strain of the virus. However, there are multiple genotypes and serotypes of IBV strains, and co-infection of multiple serotypes is common in the field. Hence, it is necessary to observe the inhibitory effect of PEO on other serotypes of IBV strains or PEO on the simultaneous challenge of multiple serotypes of IBV strains in the future, in order to explore a potentially effective alternative approach that can combat all serotypes of IBV strains.

## Conclusion

In conclusion, the PEO mixture of CA and GML had remarkable inhibition against IBV and the PEO acts by inhibiting virus multiplication and promoting immune function, suggesting that the PEO has great potential as a novel anti-IBV agent for the inhibition of IBV infection. Our study also provides a reference for effective treatments and prevention of other animal coronaviruses. Further studies are needed to better understand the likely mechanism of this inhibition, underlying immune mechanisms causing these effects, and to assess the ability of PEO to provide a safe and effective alternative method against avian IBV in commercial production facilities.

## Methods

### Virus strain and PEO

The pathogenic IBV strain M41 (Lot number AV1511) was purchased from the China Institute of Veterinary Drug Control and propagated in specific pathogen free (SPF) 10-day-old embryonating chicken eggs (Beijing Merial Vital Laboratory Animal Technology Co., Ltd., Beijing, China). The 50% tracheal organ culture infection dose (TOC-ID_50_) of IBV M41 strain was examined and calculated according to the Reed-Muench method [56]. A synergistic blend of PEO, which included CA (5%) and GML (95%), designated as Jin-Jing-Zi, was provided from Guangzhou Nasheng Biological Co., Ltd., Guangdong, China. Shuanghuanglian Oral Liquid with honeysuckle, scutellaria, and forsythia as main components, the positive drug control, was obtained from Beijing Ruixianong Technology Development Co., Ltd., Beijing, China. 1 mL of the positive drug is equivalent to 1.5 g of the original drug, and 1 mL of plant essential oil used in this experiment contains 0.2 g of active ingredients.

### Animal experimental design

140 3-day-old Yellow feather broiler chicks, purchased from Guangxi Fufeng Agricultural and Animal Husbandry Group Co., Ltd. (Nanning, China), were randomly assigned to seven groups of 20 birds each, namely blank control, challenge control, prevention, positive drug, PEO-L, PEO-M, and PEO-H groups (Table 2). The birds in the challenge control group were infected but untreated PEO. The birds in the blank control group were uninfected and untreated PEO. The whole experimental period lasted 20 days. At 3-day-old, the PEO was administered to the birds of the prevention group (1.5 mL/L water) 5 days before challenge. At 8-day-old, the birds in all groups, except the blank control group, were challenged with nasal-ocular 10^8^ TOC-ID50 of the IBV M41 strain in 0.2 mL. And all birds in the other PEO dose groups and the positive drug group were administered drugs by drinking water when more than half of the chickens showed clinical signs of the disease (13 days old). The PEO-L, PEO-M, and PEO-H groups were given orally 0.5 mL/L, 1.0 mL/L, and 2.0 mL/L PEO respectively. The positive drug group received only Shuanghuanglian Oral Liquid at 0.7 mL/L does following the manufacturer’s instructions. All chicks were given water and feed ad libitum from 8 to 12 days old. Birds in the PEO-L, PEO-M, PEO-H, and positive drug groups were given drugs in drinking water for 5 consecutive days from 13 to 17 days old. All birds were placed into molded plastic separate isolators and fed ad libitum. The birds in challenge groups and the black control group were fed in two separate and nonadjacent rooms to avoid cross-infection.

**Table 2:** Animals grouping and treatment

Groups	Treatment		
	D3-D7	D8	D13-D17
Blank control	-	-	-
Challenge control	-	IBV challenge	-
Prevention	PEO (1.5 mL/L)	IBV challenge	-
Positive drug	-	IBV challenge	positive drug (0.7 mL/L)
PEO-L	-	IBV challenge	PEO (0.5 mL/L)
PEO-M	-	IBV challenge	PEO (1.0 mL/L)
PEO-H	-	IBV challenge	PEO (2.0 mL/L)

### Evaluation of clinical symptoms and efficacy

After challenge all birds in the trial groups were daily monitored the clinical signs, including mental state, feeding, and drinking water state, head-shaking, gasping, sneezing, coughing, and so forth for 12 days (Table 3). Furthermore, the reduction of symptoms scores was recorded following administration (Table 4) [57]. The cure rates and protection rates were calculated to evaluate the efficacy of PEO on IB on the 7th day after drug administration. Cure rate = number of cured animals/total number of diseased animals × 100%; Protection rate = number of non-diseased animals/total number of animals × 100%.

**Table 3:** The score criteria for clinical evaluation

Inspection item	Clinical symptom	Score
Mental state	Lively and active, with bright eyes	0
	Listlessness and eyes are sightless	2
	Depression, fear of cold and cluster together	4
Feeding condition	Appetite is exuberant and food is snatched	0
	Loss of appetite, leftovers	2
	Lost appetite, hardly ate	4
Status of drinking water	Act leisurely and drink easily	0
	Hardly drinking water	4
Head-shaking phenomenon or not	No head shaking	0
	Mild head shaking	2
	Frequent head shaking	4
Cough condition	No cough symptoms	0
	Slight coughing	2
	Frequent coughing	4
Rale in trachea or not	No tracheal rale	0
	A slight rale in the trachea	2
	Tracheal heavy-tone	4
Breath state	Normal breathing	0
	Shortness of breath	2
	Dyspnea, mouth breathing	4

**Table 4:** The criteria for judging curative effect

Results	Disappearance of symptoms	Reduction of symptom score
Recure	Symptoms disappear or basically disappear	Symptom score reduction ≥ 95%
Significant effect	The symptoms improved significantly	Symptom score reduction ≥ 70%, < 95%
Turn for the better	The symptoms have improved	Symptom score reduction ≥ 35%, < 70%
Invalid	No significant improvement in symptoms, or even aggravation	Symptom score reduction ≤ 35%

### Detection of virus loads by real-time quantitative PCR

After 2 and 5 days of drug withdrawal, 5 birds from each group were euthanized by cervical dislocation to collect the trachea and kidney tissue samples. RNA in tracheas and kidneys was extracted using a commercial AxyPrep™ multisource total RNA miniprep kit (Corning Biotech, Suzhou, China). The viral RNA levels were assessed by qPCR targeting the N gene as our previous description [50]. Absolute quantification analyses were used to determine the absolute copy numbers of IBV RNA and the levels of mRNA expression of target cytokine genes. A forward primer (5′-CAGAAGAAGGGCTCTGCATTAC-3′) and a reverse primer (5′-AGGTTGAGCATTGCCGTAACAC-3′) were used for amplifying the IBV strain M41. The PCR system included 10 µL SYBR Green PCR mix (Vazyme Biotech Co., Ltd, Nanjing, China); 0.4 µL 10 pmol/µL upstream and downstream primers respectively; 2 µL cDNA templates, and 7.2 µL water. The PCR was performed at 95℃ for 30 s; 40 cycles of 95℃ for 5 s, 60℃ for 30 s; 60℃ for 20 s. The IBV RNA copy number was calculated according to the standard curve using the cycle threshold (Ct).

### Measurement of immune organs index

At 2 and 5 days of drug withdrawal, five chickens per group were individually weighed to determine body weight. The spleen, thymus, and bursa of Fabricius of five birds from each group were then collected and weighed separately to calculate relative organ weights, which were represented as immune organ indices. Organ index = organ weight (milligram)/body weight (gram) [58].

### Detection of specific anti-IBV antibody

Serum samples were harvested from the birds before administration (5 dpc), on days 3 (8 dpc) and 5 (10 dpc) of administration, the 2nd (12 dpc), and 5th day (15 dpc) after drug withdrawal for the antibody titer assay. Total serum IBV-specific antibody was tested using an indirect enzyme-linked immunosorbent assay (ELISA) kit (IDEXX Laboratory, Inc., Westbrook, ME, USA) according to the manufacturer’s instructions.

### Measurement of IFN-γ, IL-4, and IL-6 in serum

Serum samples were collected before administration, on the 3rd and 5th day after administration, days 2 and 5 of drug withdrawal. The levels of anti-inflammatory cytokine IFN-γ and IL-4, as well as proinflammatory cytokine IL-6 concentrations, were evaluated by ELISA Kits (Jiubang Biotechnology Co., Ltd., Quanzhou, China) following the instructions.

### Statistical analysis

The replicate (*n* = 5) has been regarded as an experimental unit. GraphPad Prism 8 and Microsoft Excel were used for the statistical analysis of all data, including the determination of the calculation of the means and the geometric mean with 95% CI. The clinical signs score curve was obtained using GraphPad Prism 8. Statistical analyses of viral loads, organ indices, IBV-specific antibody titers, and cytokine concentrations were performed by a one-way analysis of variance (ANOVA) using SPSS 24.0 software. *Post-hoc* analyses were carried out to identify the significant difference between treatment means using Tukey’s test. *P* values less than 0.05 (*p* < 0.05) were considered significant and those less than 0.01 were considered highly significant.

## Supplementary Information

**Additional file 1:** **SupplementaryTable 1.** The clinical signs score of IBV-infected chickstreated with PEO indifferentperiods.)

**Additional file 2:** **Supplementary Table 2** .The basic diet composition and nutrient level.)

**Additional file 3:** **Supplementary Fig. 1. **Pathological changes of trachea in chicken. (A)Blank control. (B) Challenge control. (C)Prevention. (D) Positive drug. (E) PEO-L. (F) PEO-M. (G)PEO-H.)

## Data Availability

The datasets analyzed in the present study are available from the corresponding author upon request.

## Abbreviations

IBV: Infectious bronchitis virus
IB: Infectious bronchitis
PEO: Plant essential oils
CA: Cinnamaldehyde
GML: Glycerol monolaurate
PEO-L: PEO low dose
PEO-M: PEO middle dose
PEO-H: PEO high dose
qPCR: Real-time quantitative PCR
dpc: Days post-challenge
IFN-γ: Gamma interferon
IL-4: Interleukin-4
IL-6: Interleukin-6
FCoV: Feline coronavirus
TEO: Thymus vulgaris essential oil
TCID_50_: Tissue culture infectious dose 50
PEDV: Porcine epidemic diarrhea virus
RSV: Respiratory syncytial virus
ASFV: African swine fever virus
VSV: Vesicular stomatitis virus
HIV: Human immunodeficiency virus
ADV: Adenovirus
NDV: Newcastle disease virus
MDV: Marek’s disease virus
SPF: Specific pathogen free
TOC-ID_50_: 50% Tracheal organ culture infection dose
ELISA: Enzyme-linked immunosorbent assay
