# Supplementary material for: In vivo antiviral effect of plant essential oils against avian infectious bronchitis virus
Source: BMC Vet Res. 2022 Mar 7;18:90. doi: 10.1186/s12917-022-03183-x (PMC8899001; doi:10.1186/s12917-022-03183-x)
Supplement: Supplementary file 1 — Additional file 1: SupplementaryTable 1. The clinical signs score of IBV-infected chickstreated with PEO indifferentperiods.) [file 12917_2022_3183_MOESM1_ESM.docx]

**Supplementary Table 1** The clinical signs score of IBV-infected chicks treated with PEO in

different periods.

|  | Blank control | Challenge control | Prevention | Positive drug | PEO-L | PEO-M | PEO-H |
| --- | --- | --- | --- | --- | --- | --- | --- |
| Before challenge | 0 | 0 | 0 | 0 | 0 | 0 | 0 |
| After challenge | 0 | 24 | 4 | 24 | 24 | 24 | 24 |
| Day 1 of administration | 0 | 24 | 4 | 24 | 24 | 24 | 24 |
| Day 2 of administration | 0 | 24 | 4 | 18 | 18 | 18 | 18 |
| Day 3 of administration | 0 | 22 | 4 | 16 | 14 | 16 | 16 |
| Day 4 of administration | 0 | 20 | 4 | 14 | 14 | 14 | 14 |
| Day 5 of administration | 0 | 16 | 4 | 6 | 8 | 2 | 2 |
| Day 1 of withdrawal | 0 | 14 | 2 | 4 | 2 | 2 | 2 |
| Day 2 of withdrawal | 0 | 10 | 2 | 2 | 2 | 0 | 2 |
| Day 3 of withdrawal | 0 | 10 | 2 | 2 | 2 | 0 | 2 |
