# Supplementary material for: In vivo antiviral effect of plant essential oils against avian infectious bronchitis virus
Source: BMC Vet Res. 2022 Mar 7;18:90. doi: 10.1186/s12917-022-03183-x (PMC8899001; doi:10.1186/s12917-022-03183-x)
Supplement: Supplementary file 2 — Additional file 2: Supplementary Table 2 .The basic diet composition and nutrient level.) [file 12917_2022_3183_MOESM2_ESM.docx]

**Supplementary Table 2** The basic diet composition and nutrient level.

| Items | Content/% |
| --- | --- |
| Ingredients |  |
| Corn | 66.4 |
| Soyabean meal | 20.2 |
| Lard | 5.0 |
| Corn Gluten | 3.0 |
| Soybean oil | 1.0 |
| Dicalcium phosphate | 0.9 |
| Limestone | 1.2 |
| Sodium chloride | 0.3 |
| Premix^1^ | 2.0 |
| Total | 100.0 |
| Calculated nutrient levels |  |
| Apparent metabolizable energy (MJ/kg) | 13.33 |
| Crude protein | 15.03 |
| Calcium | 0.74 |
| Available phosphorus | 0.32 |
| Lysine | 0.85 |
| Methionine | 0.34 |
| Threonine | 0.68 |

1 Premix provided per kilogram of diet: vitamin A (transretinyl acetate), 8000 IU; vitamin D3 (cholecalciferol), 2200 IU; vitamin E (allrac-α-tocopherol), 8 IU; menadione, 1.5 mg; thiamin, 2.0 mg; riboflavin, 4.0 mg; vitamin B6, 2.0 mg; vitamin B12 (cobalamin), 0.02 mg; nicotinamide, 30.0 mg; [pantothenic](C:/Users/HP/AppData/Local/youdao/dict/Application/8.10.3.0/resultui/html/index.html#/javascript:;) [acid](C:/Users/HP/AppData/Local/youdao/dict/Application/8.10.3.0/resultui/html/index.html#/javascript:;), 8.0 mg; folic acid, 1.0 mg;Cu (from copper sulphate), 6.0 mg; Fe (from ferrous sulfate), 80.0 mg; Mn (from manganese sulphate), 45.0 mg; Zn (from zinc oxide), 55.0 mg; Se (from sodium selenite), 0.15 mg.
