# Supplementary material for: In vivo antiviral effect of plant essential oils against avian infectious bronchitis virus
Source: BMC Vet Res. 2022 Mar 7;18:90. doi: 10.1186/s12917-022-03183-x (PMC8899001; doi:10.1186/s12917-022-03183-x)
Supplement: Supplementary file 3 — Additional file 3: Supplementary Fig. 1. Pathological changes of trachea in chicken. (A)Blank control. (B) Challenge control. (C)Prevention. (D) Positive drug. (E) PEO-L. (F) PEO-M. (G)PEO-H.) [file 12917_2022_3183_MOESM3_ESM.docx]

On the second day and the fifth day after drug withdrawal, 5 chickens were killed in each group. It was observed that the trachea and bronchus of the challenge control group were mucous to varying degrees, and the trachea ring was also congestion, while the trachea of the other drug groups and prevention groups had no obvious lesions.


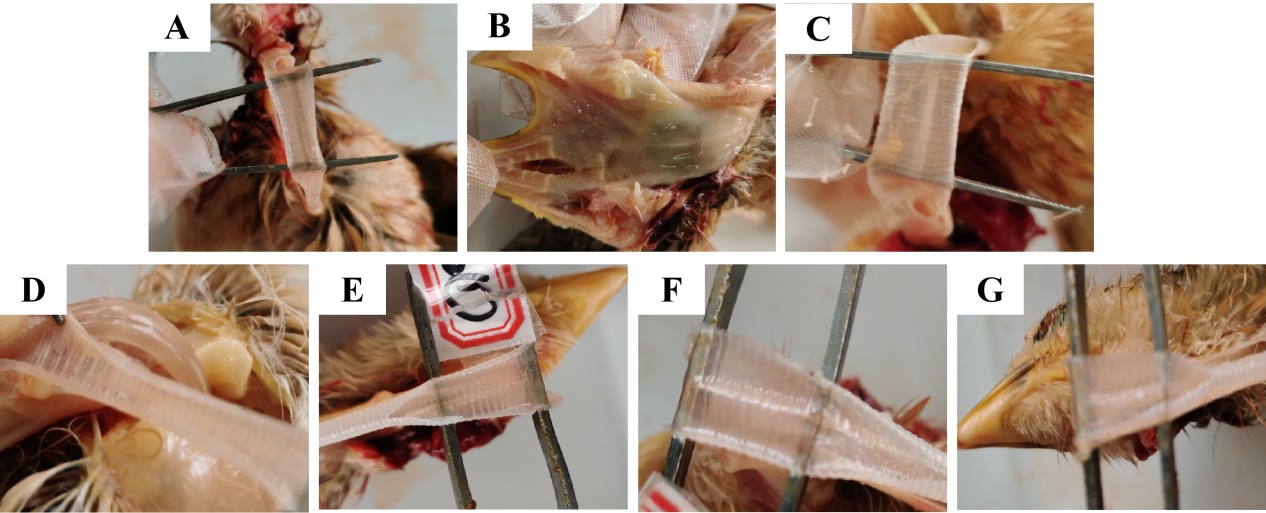


**Supplementary Fig. 1** Pathological changes of trachea in chicken. (**A**) Blank control. (**B**) Challenge control. (**C**) Prevention. (**D**) Positive drug. (**E**) PEO-L. (**F**) PEO-M. (**G**) PEO-H.
